# Sleep quality and associated factors among people who inject drugs in Iran: a nationwide survey using respondent-driven sampling

**DOI:** 10.1186/s12889-024-19368-y

**Published:** 2024-08-05

**Authors:** Rahmatollah Moradzadeh, Khosro Sadeghniiat-Haghighi, Arezu Najafi, Hamid Sharifi, Narges Abdolmohamadi, Fatemeh Hadavandsiri, Samaneh Akbarpour

**Affiliations:** 1 Department of Epidemiology, School of Health, Arak University of Medical Sciences, Arak, Iran; 2 Occupational Sleep Research Center, Baharloo Hospital, Tehran University of Medical Sciences, Tehran, Islamic Republic of Iran; 3 HIV/STI Surveillance Research Center, and WHO Collaborating Center for HIV Surveillance, Institute for Futures Studies in Health, Kerman University of Medical Sciences, Kerman, Iran; 4 Students’ Scientific Research Center, Tehran University of Medical Sciences, Tehran, Iran; 5 Non-Communicable Diseases Research Center, Endocrinology and Metabolism Population Sciences Institute, Tehran University of Medical Sciences, Tehran, Iran; 6 Sleep Breathing Disorders Research Center (SBDRC), Tehran University of Medical Sciences, Tehran, Iran

**Keywords:** Sleep quality, People who inject drugs, Respondent-driven sampling, Sleep disturbance, Injection drug use, Iran

## Abstract

**Background:**

Poor sleep quality is a significant issue among people who inject drugs (PWID). This study aimed to evaluate sleep quality and associated factors among PWID in Iran.

**Methods:**

Using respondent-driven sampling, 2,652 PWID (2,563 male) were recruited in 11 major cities in Iran between 2019 and 2020. The Pittsburgh Sleep Quality Index was utilized to measure sleep quality, and logistic regression was used to assess associations in RDSAnalyst, a software designed for respondent-driven sampling.

**Results:**

The overall prevalence of poor sleep quality was 68.4% (68.3% among males and 70.2% among females). Married PWID had higher odds of poor sleep quality (Adjusted Odds Ratio (AOR): 1.41; 95% CI: 1.05, 1.91). Lack of access to sufficient food in the past 12 months was also associated with poor sleep quality (AOR: 1.73; 95% CI: 1.17, 2.57 for sometimes having no access, and AOR: 2.95; 95% CI: 1.93, 4.52 for always having no access compared to always having access). Additionally, good self-rated health was significantly associated with lower odds of poor sleep quality (AOR: 0.19; 95% CI: 0.11, 0.31).

**Conclusion:**

Poor sleep quality is prevalent among PWID in Iran. It is recommended to mitigate the adverse effects of this issue and enhance the overall quality of life for PWID. Supportive interventions aimed at preventing and treating poor sleep quality, as well as improving overall health outcomes, are essential.

## Introduction

Poor sleep quality is a detrimental factor that contributes to individual health issues and serious medical disorders [1]. It is a significant public health and clinical matter [2] that adversely affects overall quality of life [3]. A variety of stressors, such as stigma, lack of social support, unemployment, and poverty, can negatively impact sleep quality [4]. An irregular sleep-wake rhythm is often related to a chaotic lifestyle and can result in poor sleep patterns. Poor sleep quality may be caused by various factors, including opportunistic infections, a weakened immune system, adverse effects of antiretroviral therapy (ART), HIV comorbidities, frequent substance use [5], and excessive tobacco use [6]. Poor sleep quality can lead to poorer medication adherence and worsening of disease progression.

Sleep is a critical area in the context of drug use [7]. Drug use negatively impacts sleep quality [8]. Furthermore, sleep quality and drug use have a reciprocal relationship [9]. Poor sleep quality may also be associated with the worsening development of a substance use disorder [10]. In one study, investigators reported that poorer sleep quality was linked to greater drug craving [11].

The relationship between sleep disturbances and addiction is complex, with sleep problems often serving as both a consequence and a risk factor for substance abuse [12]. Sleep disturbances, which contrast with normal mental, emotional, and physical functioning [13], play a significant role in addiction dynamics. Additionally, sleep disturbances have been identified as a potential risk factor for addiction relapse [11], highlighting the crucial need to address sleep quality in addiction recovery programs.

Sleep plays a fundamental role in mental health status [14]. Several factors influence sleep quality [15], and food insecurity is one such factor that can adversely affect it [16]. Previous studies have confirmed a positive association between poor sleep quality and food insecurity [17]. Researchers have indicated a relationship between poor sleep quality and high levels of food insecurity [18]. A study by Troxel et al. found that extreme levels of food insecurity increase the risk of both short and long sleep durations compared to the recommended 7–9 h of sleep per night [19].

A study by Guo, L., et al., revealed a U-shaped relationship between sleep duration and misuse of medical prescription drugs. Specifically, individuals with sleep durations of less than or equal to 5 h per weekday and greater than 9 h per weekday were at a higher risk for opioid or prescription drug abuse [20]. Asaad et al. found that sleep duration decreased and sleep latency increased in adults with opioid use disorders [21]. Similarly, a comprehensive review by Angarita, G. A., et al., confirmed reductions in total sleep time and increases in sleep latency [8]. Gradual abstinence from chronic opioid use has been associated with alterations in sleep parameters [22], including changes in sleep duration and latency.

Marital status has a significant impact on sleep quality, with various studies highlighting the complex relationship between marital status and sleep outcomes. The available evidence suggests that being married or cohabiting can both positively and negatively affect sleep quality [23]. For example, co-sleeping with a partner can lead to higher levels of sleep fragmentation, resulting in reduced total sleep duration [24]. Furthermore, factors such as marital transitions, separation, or divorce can be linked to greater instances of sleep disturbances. Studies emphasize the importance of considering socio-ecological models to understand the mechanisms behind sleep disturbances related to marital status [25].

Despite the potential complications arising from poor sleep quality among people who inject drugs (PWID), there has been limited research focusing on sleep quality within this population in Iran [26–28]. Understanding the sleep patterns among PWID could significantly enhance the design and effectiveness of intervention programs, given that sleep problems have been linked to relapse and concurrent psychological issues. The current study aimed to address this gap by conducting the first national study investigating the prevalence of sleep disturbances and their associated factors among PWID in Iran.

## Methods and materials

### Design and sampling method

This national cross-sectional study was conducted using data from the bio-behavioral surveillance surveys (BBSS) of Iranian PWID from July 2019 to March 2020 in 11 major cities in Iran including Tehran, Tabriz, Sari, Mashhad, Kermanshah, Lorestan, Yazd, Ahvaz, Shiraz, Kerman, and Zahedan. The cities were selected to ensure comprehensive geographic coverage and provide a representative sample of the entire country. Samples were divided based on the estimated proportion of PWID in each city. Since our study was part of the national bio-behavioral surveillance surveys of Iranian PWID, we did not calculate the sample size in this secondary analysis. As the study focused on PWID in Iran, a sleep questionnaire was administered to them. In this study, we explore the sleep information of PWID. The detailed survey methodology has been previously described [29, 30]. Since the PWID are a hidden population that cannot be reached in sufficient numbers using conventional sampling methods, a total of 2,652 PWID were recruited using respondent-driven sampling (RDS). This method allowed for multiple recruitment waves, reaching farther into social networks and enhancing sample representativeness and generalizability [31]. Participants were recruited for the study through initial recruits, also referred to as ‘seeds,’ using non-random and purposeful selection methods. These seeds were chosen to initiate the chain-referral process from various locations, including drop-in centers, shelters, Opium Maintenance Treatment (OMT) centers, voluntary counseling and testing centers, and outreach spots. This approach was designed to ensure maximum variation in the sample [32]. The study was started with 80 initial recruits who received three referral coupons to be used over a period of three weeks. These initial recruits were trained to use the coupons to recruit three peers who met the inclusion criteria: being 18 years or older, self-reporting injecting drugs in the past 12 months, holding Iranian citizenship, and possessing a valid referral coupon. The recruitment process continued until a total of 2,652 PWID were successfully recruited. Additional details can be found in a previously published article [29, 30]. Once verbal consent was obtained, gender-matched trained interviewers conducted face-to-face interviews using standard questionnaires to gather behavioral information.

### Study measures

The study collected data in two sections. The first section included demographic information, such as age group (18–29, 30–40, …, 50+), education level (illiterate, elementary, intermediate, diploma, and academic), marital status (single, married, divorced, or separated), prison stay history (yes, no), monthly income (less than $48, over $48), homelessness history (yes, no), and access to adequate food by answering the question “Which of the following sentences best describes you and your family sufficient food access in the past 12 months” (always, often, sometimes, no access). Additionally, participants self-rated their health status by answering one Likert scale question: “In general, would you say that your health status?” (Very poor, poor, moderate, good).

The second section of the study utilized the Pittsburgh Sleep Quality Index (PSQI) questionnaire, previously validated in Persian [33]. The Cronbach’s alpha coefficient of the PSQI was 0.77 for all subjects, and the corrected item–total correlations ranged from 0.30 to 0.75 for the PSQI. This questionnaire consists of 19 items.

The Pittsburgh Sleep Quality Index (PSQI) questionnaire consists of 19 items, which are grouped into seven components: sleep quality, sleep latency, sleep duration, habitual sleep efficiency, sleep disturbance, use of sleep medication, and daytime dysfunction. Each component is scored on a scale of 0 to 3, where 0 represents no difficulty and 3 represents severe difficulty. The scores of the seven components are summed to obtain a total score ranging from 0 to 21. Higher scores indicate poorer sleep quality. In this study, a total score above 5 is considered indicative of poor sleep quality [34].

### Statistical analysis

Data were described by mean and standard deviation (SD) for quantitative variables, and percentage and 95% confidence intervals (CIs) for qualitative variables. Population estimates and 95% CIs were calculated utilizing respondent-driven sampling in RDS Analyst, a software package designed for analyzing Respondent-driven sample data [35]. Bivariable and multivariable logistic regressions were conducted to assess the association between the variables studied and poor sleep quality. Variables with a P-value less than 0.2 in the bivariable model were included in the multivariable logistic regression. A P-value equal to or less than 0.05 was used to show statistically significant results. Stata and RDSAnalyst software v.12 were employed to analyze the data.

## Results

Overall, 2,652 PWID (2,563 male and 89 female) were included in the analysis. Table 1 contains the demographic characteristics of the participants. The average age of the participants was 40.4 (SD: 9.3) years. The majority of them were male (96.5%) and 37.3% had an intermediate-level education.

**Table 1:** The prevalence of sleep quality among people who inject drugs (PWID) in Iran during the years 2019–2020 based on sociodemographic characteristics

Variables		Total (%)	Population estimates in %		P-value
			Good sleep quality	Poor sleep quality	
Sex	Male	96.5	31.7	68.3	0.057
	Female	3.5	29.9	70.2	
Age (years)	18–29	8.6	46.8	53.2	0.248
	30–40	32.4	26.1	73.9	
	40–50	42.6	34.6	65.4	
	≥ 50 and more	16.5	31.9	68.1	
Education level	Illiterate	7.4	31.6	68.4	0.001
	Elementary	23.4	36.2	63.8	
	Intermediate	37.3	24.1	75.9	
	Diploma	24.7	44.7	55.4	
	Academic	7.2	34.8	65.2	
Homelessness	No	55.1	32.6	67.4	0.001
	Yes	44.9	21.6	78.4	
Monthly income*	Less than $48	89.1	28.4	71.6	0.001
	More than $48	10.9	41.8	58.2	
Marital status	Married	29.6	37.8	62.3	0.001
	Divorced or separated	34.9	26.3	73.7	
	Single	35.5	33.6	36.4	
Prison history	No	22.1	31.7	68.3	0.001
	Yes	77.9	29.5	70.5	
Access to sufficient food in the past 12 months	Always	14.5	54.6	45.4	0.001
	Often	43.7	32.5	67.5	
	Sometimes	20.3	24.4	75.6	
	No access	21.5	16.0	84.0	
Self-rated health	Very Bad	9.3	11.7	88.3	0.001
	Bad	21.7	20.5	79.5	
	Moderate	37.8	28.3	71.7	
	Good	31.2	54.8	45.2	

*$48 has been converted into 42,000 Iranian rials

Of all participants, 68.4% (CI95%: 0.67, 0.70) experienced poor sleep quality. As shown in Table 1, among male and female study participants, 68.3% of men and 70.2% of women also experienced poor sleep quality. Individuals in the 30–40 age group had a higher prevalence of poor sleep quality (73.9%), while adults in the 18–29 age group had a higher prevalence of good sleep quality (46.8%). Poor sleep quality in relation to educational level was present in 75.9% of individuals with an intermediate educational level, and 67.4% of participants with the history of homelessness experienced poor sleep quality. Participants with a monthly income of less than 48 dollars had a higher prevalence of poor sleep quality (71.6%). Poor sleep quality affected 73.7% of those who were divorced or separated. Of those with a history of incarceration, 70.5% had poor sleep quality. Participants who often do not have access to sufficient food had a higher prevalence of poor sleep quality (84%) compared to participants who had access to sufficient and diverse food in the past 12 months. Individuals who had a very poor health rating were more likely to have poor sleep quality (88.3%) compared to those with other self-rated health statuses. There were statistically significant differences between sleep quality and education level, homelessness, monthly income, marital status, incarceration, access to adequate food in the past 12 months, and self-rated health (all p-values ≤ 0.001) (Table 1).

Table 2 shows a comparison of sleep quality components in two groups. Overall, subjective sleep quality and sleep latency had higher mean scores, indicating more problems compared to other domains of sleep quality in this group of people with poor sleep quality.

**Table 2:** Comparing sleep quality components among people who inject drugs (PWID) in Iran during the years 2019–2020 with good and poor total sleep quality

Variables	Total	Good sleep quality	Poor sleep quality	P-value
	Mean (SD)			
Subjective sleep quality	1.52 (0.72)	0.97 (0.31)	1.77 (0.71)	0.001
Sleep latency	1.18 (0.95)	0.46 (0.61)	1.48 (0.91)	0.001
Sleep duration	0.76 (0.97)	0.23 (0.47)	0.95 (1.03)	0.001
Sleep efficiency	0.59 (1.00)	0.07 (0.27)	0.67 (0.98)	0.001
Sleep Disturbances	1.11 (0.63)	0.62 (0.50)	1.31 (0.57)	0.001
Use of Sleeping Medication	0.80 (1.04)	0.11 (0.31)	1.09 (1.09)	0.001
Daytime Dysfunction	1.29 (0.99)	0.45 (0.54)	1.62 (0.92)	0.001
Total	7.11 (3.81)	2.89 (1.03)	8.89 (3.08)	0.001

Gile’s SS Estimate was used for all variables

Table 3 shows the results of the bivariable logistic regression for the relationship between sleep quality and the study variables. The results of the bivariate analysis indicate statistically significant associations between homelessness (Odds Ratio [OR] = 1.50, 95% CI: 1.23–1.83), being divorced or separated (OR = 2.15, 95% CI: 1.66–2.79), being single (OR = 1.31, 95% CI: 1.02–1.67), incarceration (OR = 1.83, 95% CI: 1.46–2.30), and access to sufficient food in the past 12 months. Specifically, often having access to sufficient food (OR = 1.88, 95% CI: 1.36–2.61), sometimes having no access to sufficient food (OR = 3.24, 95% CI: 2.28–4.61), and always having no access to sufficient food (OR = 7.03, 95% CI: 4.85–10.19) were associated with poor sleep quality.

**Table 3:** Factors associated with poor sleep quality in bivariate logistic regression model among PWID

Variables		OR (95% CI)	p value
Sex	Male		
	Female	2.04 (0.96, 4.32)	0.062
Age (years)	Lower than 30	Reference	
	30–40	1.12 (0.79, 1.59)	0.528
	40–50	1.17 (0.83, 1.65)	0.373
	50 and more	1.49 (0.98, 2.26)	0.061
Education level	Illiterate	Reference	
	Elementary	0.82 (0.52, 1.27)	0.373
	Intermediate	0.84 (0.56, 1.28)	0.418
	Diploma	0.61 (0.40, 0.93)	0.022
	Academic	0.36 (0.20, 0.63)	0.001
Homelessness	No	Reference	
	Yes	1.50 (1.23, 1.83)	0.001
Monthly income*	Less than $48	Reference	
	More than $48	0.47 (0.35, 0.63)	0.001
Marital status	Married	Reference	
	Divorced or separated	2.15 (1.66, 2.79)	0.001
	Single	1.31 (1.02, 1.67)	0.031
History of prison	No	Reference	
	Yes	1.83 (1.46, 2.30)	0.001
Access to sufficient food in past 12 month	Always	Reference	
	Often	1.88 (1.36, 2.61)	0.001
	sometimes	3.24 (2.28, 4.61)	0.001
	No access	7.03 (4.85, 10.19)	0.001
Self-rated health	Very bad	Reference	
	Bad	0.88 (0.54, 1.43)	0.607
	Moderate	0.68 (0.44, 1.06)	0.086
	Good	0.16 (0.11, 0.25)	0.001

*$48 has been converted into 42,000 Iranian rials.

*OR: odds ratio, CI: Confidence interval

Table 4 demonstrates the results of multivariable logistic regression: divorced or separated participants had a higher odds for poor sleep quality (Adjusted OR [36] = 1.41, 95% CI: 1.05, 1.91). In addition, individuals who sometimes had no access to sufficient food in the past 12 months (AOR = 1.73, 95% CI: 1.17–2.57) and those who had no access to sufficient food in the past 12 months (AOR = 2.95, 95% CI: 1.93–4.52) had higher odds of poor sleep quality compared to those who always had access to sufficient food. Moreover, the odds of poor sleep quality among those who reported their health as good were lower than among those who reported their health status as very poor (AOR = 0.19, 95% CI: 0.11–0.31).

**Table 4:** The factors associated with poor sleep quality in multiple logistic regression model among PWID

Variables		AOR (95% CI) ^*^	*p* value
Marital status	Married	Reference	
	Divorced or separated	1.41 (1.05, 1.91)	0.024
	Single	1.16 (0.88, 1.52)	0.299
Access to sufficient food in the past 12 months	Always	Reference	
	Often	1.29 (0.90, 1.84)	0.164
	Sometimes	1.73 (1.17, 2.57)	0.006
	No access	2.95 (1.93, 4.52)	0.001
Self-rated health	Very bad	Reference	
	Bad	0.65 (0.38, 1.11)	0.119
	Moderate	0.69 (0.41, 1.14)	0.148
	Good	0.19 (0.11, 0.31)	0.001

*AOR: Adjusted odds ratio, CI: Confidence intervals

## Discussion

Previous studies have consistently shown that sleep problems often precede drug use, dependence, and relapse [37]. In fact, the early onset of drug use has been linked to poorer sleep quality later in life [38]. In the present study, we investigated sleep quality and its predictive factors among PWID in Iran using RDS. The results indicated that poor sleep quality was more prevalent among women than men, although the difference was not statistically significant, which is consistent with previous reports. A systematic review and meta-analysis by Kocevska, D., et al. [39], found sex differences in sleep parameters during adulthood. Insomnia was more common in women than men (19% versus 12%), with women experiencing longer total sleep duration but lower sleep efficiency. Another study confirmed the finding that insomnia was more common in women than men [40]. Social, psychological, and biological factors have been cited as explanations for the more frequent sleep problems in women [41].

In our study, we discovered that people who inject drugs (PWID) who sometimes had access to adequate food and those who had no access to adequate food in the past 12 months experienced poor sleep quality. Specifically, the prevalence of poor sleep quality among PWID who never had access to sufficient food in the past 12 months was 88.3%.

In our study, we observed that while various domains of sleep quality were impacted, sleep disturbance emerged as particularly significant in individuals with poor sleep quality. The mean score of sleep disturbance was notably higher in those experiencing poor sleep quality compared to those with good sleep quality. Given the profound impact of sleep disturbance on addiction recovery and overall well-being, interventions targeting sleep quality are imperative in addiction treatment programs.

The results of the present study support previous findings, showing that mean sleep latency was higher in individuals with poor sleep quality than in those with good sleep quality. In contrast, mean sleep duration was higher in individuals with PWID who had poor sleep quality than in individuals with good sleep quality [8, 21, 22].

The findings of our study revealed that PWID who rated their health as good were less likely to have poor sleep than participants who rated their health as very poor. This suggests a potential correlation between self-perceived health status and sleep quality among PWID. One study found that HIV-infected individuals who use drugs, particularly drug addicts, have significantly lower self-rated quality of life [42]. This study further emphasizes the multifaceted challenges faced by this population. These challenges not only include health risks associated with drug use and HIV infection but also extend to their quality of life and well-being.

In this work, marital status had a major effect on poor sleep quality. Overall, marital status plays a crucial role in sleep quality, and further research is needed to explore the complex interplay between marital factors and sleep outcomes.

In this study, national data on PWID was utilized. This marks the first time that national data has been used to represent the sleep quality status of PWID in the country, which is a significant strength of the study. However, the study also had its limitations. The study relied on self-reported data, making the results susceptible to misclassification. Another limitation of the current study is the measurement of adequate food and health rate, which we assessed using a single question and used as a proxy variable. It might be better to conduct more accurate measurements in future studies [43]. Furthermore, due to the cross-sectional design of the nationwide study, we can only assess the association and relationship of sleep quality and opioid use; however, causality cannot be determined. We acknowledge that association between two variables is mutual.

## Conclusion

This paper provides valuable insights into the sleep quality of PWID. It was observed that PWID with poor sleep quality experienced more sleep problems, with the exception of subjective sleep quality and sleep efficiency. Poor sleep quality was more common among those who had difficulties with access to sufficient food, a low level of health rate, and divorced and separated participants. It is estimated that poor sleep quality is prevalent among PWID in Iran. It is recommended to mitigate the adverse effects of this issue and enhance the overall quality of life for PWID. Supportive interventions aimed at preventing and treating poor sleep quality, as well as improving overall health outcomes, are essential.

## Data Availability

The datasets used and/or analyzed during the current study are available from the corresponding author upon reasonable request.
