# Correction: Sleep quality and associated factors among people who inject drugs in Iran: a nationwide survey using respondent-driven sampling

**DOI:** 10.1186/s12889-024-19807-w

**Published:** 2024-08-28

**Authors:** Rahmatollah Moradzadeh, Khosro Sadeghniiat-Haghighi, Arezu Najafi, Hamid Sharifi, Narges Abdolmohamadi, Fatemeh Hadavandsiri, Samaneh Akbarpour

**Affiliations:** 1 Department of Epidemiology, School of Health, Arak University of Medical Sciences, Arak, Iran; 2 Occupational Sleep Research Center, Baharloo Hospital, Tehran University of Medical Sciences, Tehran, Islamic Republic of Iran; 3 HIV/STI Surveillance Research Center, and WHO Collaborating Center for HIV Surveillance, Institute for Futures Studies in Health, Kerman University of Medical Sciences, Kerman, Iran; 4 Students’ Scientific Research Center, Tehran University of Medical Sciences, Tehran, Iran; 5 Non-Communicable Diseases Research Center, Endocrinology and Metabolism Population Sciences Institute, Tehran University of Medical Sciences, Tehran, Iran; 6 Sleep Breathing Disorders Research Center (SBDRC), Tehran University of Medical Sciences, Tehran, Iran

**Correction: BMC Public Health (2024) 24:2119**

10.1186/s12889-024-19368-y

During the publication process 2 errors were introduced in the author names and affiliation. The incorrect and correct information is shown in the below table. The original article has been updated.

**Table:** 

Incorrect	Correct
Khosro Sadeghniiat Haghigh Samaneh Akbarpour^5,6^	Khosro Sadeghniiat-Haghighi Samaneh Akbarpour^5,6^
^5^ Sleep Breathing Disorders Research Center (SBDRC), Tehran University of Medical Sciences, Tehran, Iran ^6^ Non-Communicable Diseases Research Center, Endocrinology and Metabolism Population Sciences Institute, Tehran University of Medical Sciences, Tehran, Iran	^5^ Non-Communicable Diseases Research Center, Endocrinology and Metabolism Population Sciences Institute, Tehran University of Medical Sciences, Tehran, Iran ^6^ Sleep Breathing Disorders Research Center (SBDRC), Tehran University of Medical Sciences, Tehran, Iran

